# Sociodemographic and health status differences in delaying medical care during the COVID-19 pandemic among older adults: findings from the Health and Retirement Study

**DOI:** 10.1186/s12889-022-14118-4

**Published:** 2022-09-10

**Authors:** Mateo P. Farina, Jennifer A. Ailshire

**Affiliations:** Leonard Davis School of Gerontology, University of Southern California, Los Angeles, CA 90089 USA

**Keywords:** Delaying health care, Older adults, COVID-19, Doctor visits

## Abstract

**Background:**

During the COVID-19 Pandemic, adults in the United States reported delaying medical care, which may be tied risk of infection and local policies limiting appointment. Some populations may have been more likely to delay care than others, leading to other forms of health inequality during this period. To-date there is little research on delayed care among U.S. older adult. We determine the prevalence of delayed medical care among older adults and investigate sociodemographic and health status inequalities in delaying health care.

**Method:**

We used data from the first public release of the nationally representative Health and Retirement Study COVID-19 Subsample (*N* = 3006). Using logistic regression, we assessed whether differences in delaying health care varied by age, sex, race/ethnicity, education, self-rated health (SRH), and having any Activity of Daily Living (ADL) limitation. We also conducted additional analysis that evaluated differences in delaying care by two care subtypes: doctor and dental care visits.

**Results:**

About 30% of U.S. older adults reported delaying care with the most common types of delayed care being dental or doctor visits. Adults ages 75 and older were less likely to delay care, while women, college educated, and those with poor SRH, and any ADL limitations were more likely to delay care.

**Conclusions:**

Nearly one-third of older adults delayed care during the COVID-19 pandemic. The increased likelihood of delayed care among people with worse health suggests that there may be longer-term impacts on the health care system and population health from the COVID-19 pandemic, and may contribute to health inequalities in the near future.

**Supplementary Information:**

The online version contains supplementary material available at 10.1186/s12889-022-14118-4.

## Background

The COVID-19 Pandemic has had profound effects on the health and well-being of the U.S. population. As of July 6, 2021, 44.8 million Americans have tested positive for COVID and 723,000 have died [1]. This crisis has upended the healthcare system and may have long term consequences for health of the population. Specifically, there is a growing concern on the health consequences of delaying medical care during COVID [2, 3], especially among older adults who may be most impacted by delaying care. 4 in 10 US adults 18+ have delayed or avoided routine or non-emergency care, either from personal choice or limited availability of doctors’ appointments [4], though little is known about how many older adults delayed care. Current information is now emerging that delaying care during COVID may have exacerbated dental and health problems [5]. In particular, a major concern for medical professionals has been the advancement of diabetes and fast-growing cancers (i.e., breast and colon cancer) that could be better managed and treated at the outset of the condition [6]. Researchers have predicted that colorectal and breast cancer deaths will increase by 10,000 over the next 10 years because of COVID 19’s impact on cancer care [7]. Additionally, delaying care may negatively impact the health care system in two ways: 1) limited access because the demand from people returning to using health care far exceeds availability and as result delays screening for weeks or months and 2) more advanced chronic conditions may require more intensive treatments further pulling on medical resources. The next few months and years will reveal the full extent of COVID on the health of the United States and the strain it may cause on the health care system.

We used data on adults ages 55 and older from the COVID-19 subsample of the HRS, a nationally representative sample of older adults, to investigate sociodemographic and health status differences in delaying medical care from June 2020 to September 2020. Prior research has shown that delaying medical care was highly prevalent in the US adult population due to changes in individual behaviors to mitigate COVID transmission risk as well as limited access to healthcare facilities [4]. In this study, we built on prior work by providing a clearer understanding of the differences in delaying medical care among older adults during the COVID-19 Pandemic. While several studies have documented widespread inequality in COVID-related health (e.g., infection rates and mortality), less attention has been given to other health inequalities such as delaying care. Demographic and health status characteristics may impact health inequalities in delaying care, especially when considering differences in access, risk, and other stratifying forces. Specifically, in this study, we evaluate differences delaying care by age, sex, race/ethnicity, education, self-rated health, and disability status, which are widely known to impact health inequality.

## Methods

### Data

We used data from the November Release of 2020 Health and Retirement Survey COVID-19 subsample (HRS). The HRS is a nationally representative longitudinal survey of older adults 50+ in the United States. The COVID-19 subsample of the HRS consisted of a 50% random sample of households who were originally scheduled to be interviewed for the 2020 HRS Core Wave. Data from the first half of the COVID-19 Subsample, which was interviewed between June and September, are publicly available. Interviews were conducted by phone with 3266 respondents, representing a response rate of 62%. Respondents lived either in the community or in a nursing home.

The analytical sample consisted of 3006 older adults ages 55 and older who had no missing data, approximately 3% of the sample was dropped.

### Variables

#### Delayed care

To assess delayed care, respondents were asked: “Since March 2020, was there any time you needed medical care or dental care, but delayed getting it, or did not get it at all?” Those who answered yes were asked: “What type of care was delayed …” followed by “Surgery?”, “Seeing the doctor?”, “Filling a prescription?”, and “Dental Care?”. Each type of care was asked separately. Based on these individual questions, we created 5 dichotomous indicators for: 1) any delayed care; 2) delayed seeing the doctor (telemedicine counted as seeing a doctor); 3) delayed filling a prescription; 4) delayed dental care; and 5) delayed surgery.

#### Demographics and health status covariates

In order to evaluate social and health status differences, we created several demographic and health covariates. Measures included age (55–74, 75+), sex (male, female), self-reported race/ethnicity (non-Hispanic white, non-Hispanic Black, Hispanic, and other), educational attainment (less than high school, generalized education diploma (GED) or high school completion, some college, and college degree), self-rated health (excellent, very good, good vs. poor, fair), and whether or not the respondent had any limitations in activities of daily living limitation (yes vs. no).

#### Analysis

We used logistic regression models to examine associations delaying any care, delaying dental care, and delaying seeing a doctor. We did not evaluate delaying filling a prescription or delaying surgery because the number of cases was too small to provide statistically reliable insight. We report unadjusted and adjusted odds ratios from logistic regression models. Sample weights are used in all analyses to adjust for sampling probability and survey non-response. Analyses were conducted using STATA 15.1.

## Results

The weighted descriptive statistics for the sample are reported in Table 1. The respondents were 69.33 years of age on average. Women constituted 53% of the sample. Non-Hispanic Whites composed the largest group (73.3%) followed by non-Hispanic Blacks (10.6%), Hispanics (10.4%), and non-Hispanic Others (5.7%). Over half of the respondents had college education: some college (27.52%) and bachelor’s or more (30.71%). 25.3% of the respondents reported having poor self-rated health. 12.6% reported having any ADL limitation.

**Table 1:** Weighted Sample Characteristics, HRS COVID Subsample (November 2020 Release), Age 55+ (*N* = 3006)

	Mean (s.d.)	Range	%
**Age**	69.33 (10.01)	[55–101]	
**Female**			53%
**Race**			
Non-Hispanic Whites			73.3%
Non-Hispanic Blacks			10.6%
Hispanics			10.4%
Non-Hispanic Others			5.7%
**Education**			
Less than High School			11.4%
High School Graduate			30.4%
Some College			27.5%
Bachelor’s or More			30.7%
**Poor Self-Rated Health**			25.3%
**Has at any ADL limitation**			12.6%

*s.d.*  Standard deviation

**Fig. 1:**
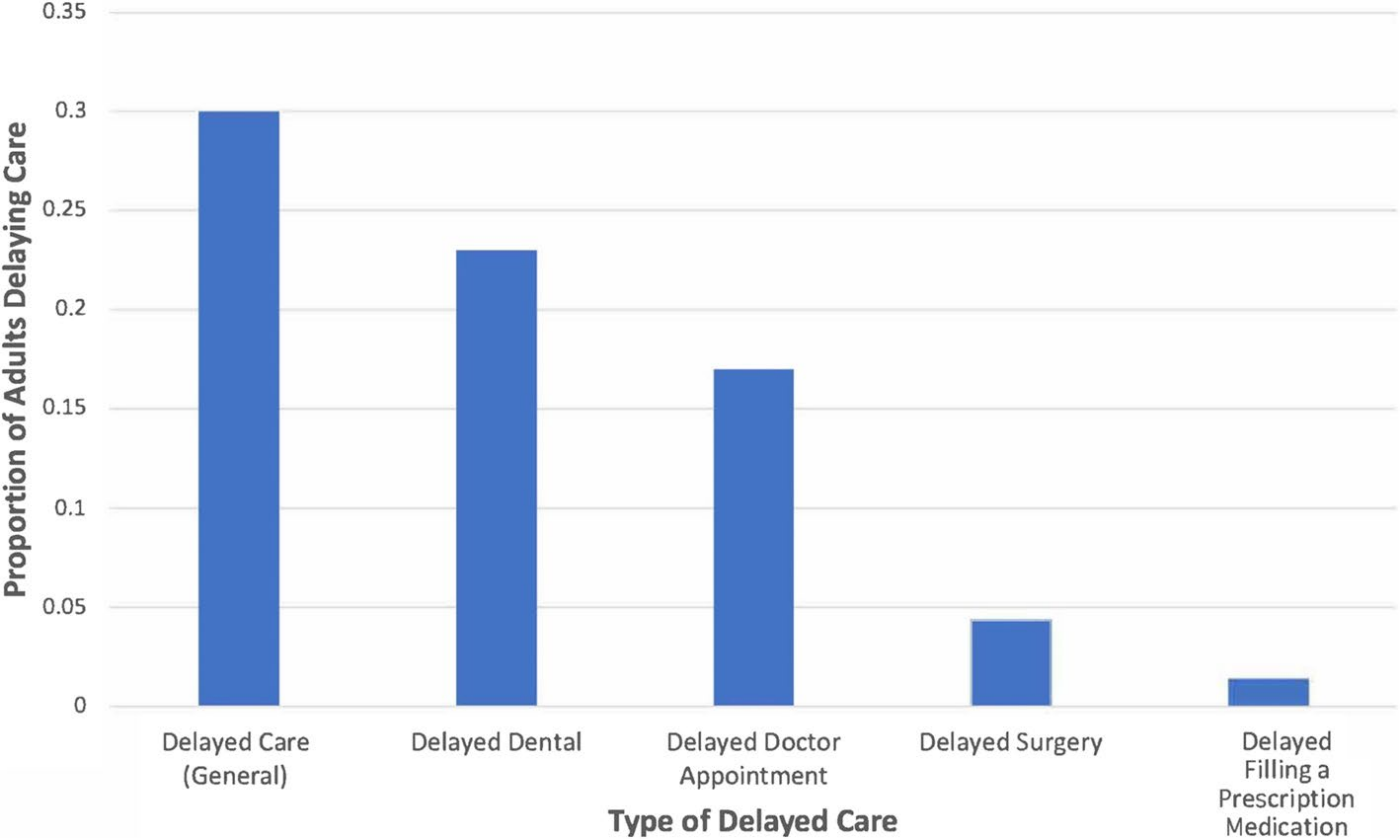
Proportion of older adults delaying care and by care type (HRS COVID Subsample June 2020 to September 2020)

The weighted prevalence of each type of delayed care are shown in Fig. 1. About 30% of respondents reported delaying care. The largest type of delayed care was dental (23%), followed by doctor visits (17%). Very few people reported delaying surgery or obtaining medication (4.4 and 1.4%, respectively).

We estimated separate logistic regressions for delayed any care, delayed dental care, and delayed medical care to determine if delayed care differed by demographic, socioeconomic, and health characteristics. We reported odds ratios and 95% confidence intervals from unadjusted and fully adjusted models. Odds ratios lower than 1 indicated lower likelihood of delaying care, whereas odds ratios greater than 1 indicated greater likelihood to delay care. These are shown in Table 2.

**Table 2:** Odds Ratio from Logistic Regression Models Predicting Delayed Care, Delayed Doctor Visit, and Delayed Dental Among Older Adults 54+ During the COVID-19 Pandemic (HRS COVID Subsample 2020, *N* = 3006)

	Delayed Care (General)				Delayed Dental				Delayed Doctor			
	Unadjusted		Adjusted		Unadjusted		Adjusted		Unadjusted		Adjusted	
	OR	CI	OR	CI	OR	CI	OR	CI	OR	CI	OR	CI
**Age 75+ (Reference:** 55**–74**	0.57***	(0.45,0.71)	0.55***	(0.43,0.70)	0.53***	(0.41,0.69)	0.54***	(0.41,0.71)	0.57***	(0.43,0.77)	0.54***	(0.40,0.73)
**Women (Reference: Men)**	1.27*	(1.02,1.57)	1.30*	(1.04,1.62)	1.16	(0.91,1.48)	1.20	(0.94,1.53)	1.23	(0.94,1.61)	1.25	(0.95,1.65)
**Race/Ethnicity (Reference: NH Whites)**												
Non-Hispanic Blacks	1.01	(0.77,1.33)	1.00	(0.75,1.32)	0.88	(0.65,1.20)	0.94	(0.68,1.30)	0.87	(0.63,1.19)	0.80	(0.57,1.12)
Hispanics	0.78	(0.57,1.08)	0.84	(0.59,1.18)	0.59**	(0.40,0.85)	0.73	(0.49,1.08)	0.91	(0.62,1.34)	0.88	(0.59,1.30)
Non-Hispanic Others	0.92	(0.55,1.51)	0.76	(0.46,1.28)	1.10	(0.65,1.86)	0.92	(0.54,1.58)	1.05	(0.57,1.94)	0.87	(0.47,1.61)
**Education (Reference: Less than High School)**												
High School or GED	1.19	(0.83,1.71)	1.23	(0.85,1.79)	1.75*	(1.14,2.69)	1.60*	(1.03,2.47)	0.86	(.55,1.33)	0.89	(0.57,1.39)
Some College	1.40	(0.98,2.01)	1.43	(0.98,2.10)	1.99**	(1.30,3.04)	1.78*	(1.14,2.78)	1.14	(0.74,1.75)	1.20	(0.77,1.86)
Bachelor’s Degree and Above	1.98***	(1.38,2.83)	2.22***	(1.51,3.26)	3.23***	(2.13,4.91)	2.97***	(1.91,4.64)	1.44	(0.94,2.21)	1.67*	(1.07,2.62)
**Poor Self Rated Health (Reference: Good Self-Rated Health)**	1.31*	(1.03,1.67)	1.43*	(1.09,1.88)	0.91	(0.70,1.20)	1.02	(0.75,1.39)	1.53**	(1.14,2.04)	1.56**	(1.11,2.20)
**Has an ADL limitation**	1.56**	(1.17,2.08)	1.69**	(1.24,2.32)	1.35	(.98,1.85)	1.76**	(1.24,2.50)	1.80***	(1.29,2.52)	1.84**	(1.26,2.68)

*p < .05, **p < .01, ***p **< .001**

For any care, adults 75 and older were less likely to delay care than adults 55–74 (OR 0.57; 95% CI 0.45–0.71). Women were more likely to delay care than men (OR 1.27; 95% CI 1.02–1.57). There were no statistically significant differences by race/ethnicity. Respondents with a bachelor’s degree were more likely to delay care (OR 1.98; 95% CI 1.38–2.83). Respondents who reported poor health (OR 1.31; 95% CI 1.03–1.67) and those who had difficulty with at least one ADL limitation were more likely to delay care (OR 1.56; 95% CI 1.17–2.08). The adjusted odds for delayed care showed the same patterns as unadjusted odds.

Next, we evaluated characteristics associated with delaying care for the two most common categories. For dental care, we found that adults 75+ were less likely to delay care (OR .53; 95% CI .41–.69). We found no sex differences. Hispanics were less likely than non-Hispanic Whites to delay dental care (OR .59; 95% CI .4–.85). Respondents with a high school diploma or greater were more likely to delay care: high school or GED (OR 1.75; 95% CI 1.14–2.69), some college (OR 1.99; 95% CI 1.3–3.04), and bachelor’s degree or greater (OR 3.23; 95% CI 2.13–4.91). There was no difference in SRH categories. The adjusted model differed slightly: Hispanics were no longer statistically different from Non-Hispanic Whites and having an ADL limitation was associated with a greater likelihood of delaying care (OR 1.76; 95% CI 1.24–2.5).

Lastly, for delayed doctor care, we found some differences across covariates. In the unadjusted models, adults 75+ were less likely to delay care (OR .57; 95% CI .43–.77). We found no differences across sex, race/ethnic groups, or education. People with poor SRH were more likely to delay care (OR 1.53; 95% CI 1.14–2.04), and people with an ADL limitation were more likely to delay care (OR 1.8; 95% CI 1.29–2.52). The adjusted models showed similar patterns. The only difference between the unadjusted and adjusted models was that bachelor’s degree holders were more likely to delay care in the adjusted models (OR 1.67; 95% CI 1.07–2.62).

We also performed supplementary analysis evaluating differences in delaying care by other potential confounders related to healthcare utilization such as frequency of doctors’ visits prior to the pandemic and cardiometabolic health conditions (See Table S1). Cardiometabolic health conditions (diabetes, heart disease, stroke, and high blood pressure) were not associated with delaying medical care. We also did not observe an association between the number of cardiometabolic conditions and delaying medical care. Similar to the association found for SRH and ADL limitations, we found that increased doctor visit frequency in 2018 was positively associated with delaying medical care. This measure was not included in full analysis because it would lead to a significant reduction in the analytical sample size.

## Discussion

In this study we determined the prevalence of delayed care among older adults and the demographic, socioeconomic, and health factors associated with delayed care. While delaying health care during the first 6 months of the COVID-19 pandemic was highly prevalent among older adults, the type of delayed care varied. Dental and doctor appointments were frequently delayed, while filling a prescription and surgery were rarely delayed. We also observed some variation in patterns of delaying by sociodemographic and health status. Differences in delaying care by type and group may have significant implications for population health in the near future, which will most likely impact health inequalities.

The risk in delaying medical care was unevenly distributed across the population. Notably, compared to men, women were more likely to delay medical care. This finding is in line with findings from studies prior to pre-COVID 19 [8]. It is concerning that women delayed medical care during COVID-19 because prior studies have found that older women have greater health needs than men [9]. Therefore, while unmet health needs are a concern for any population group, unmet care for women during this period could have implications for diagnosis, treatment, and overall care related to chronic conditions that will impact sex inequality in health in the future.

Additionally, adults with greater levels of education were likely to delay care. This finding contradicts earlier studies that found delayed medical care tied strongly to economic resources, since adults with greater levels of education have larger socioeconomic resources [10]. Delays in medical care among adults with greater levels of education may be reflective of care needs (well-educated adults may have delayed routine appointments or elective/nonessential care rather than acute care) and/or combined with risk assessment [11, 12].

Differences in delayed care by health status are concerning. We found that older adults who reported poor self-rated health or had an ADL limitation were more likely to delay care. One potential explanation may be that older adults with pre-existing conditions may need more frequent health visits; therefore, adults with poor health status may have been more likely to experience delayed care due to mandatory appointment cancelations that occurred at the beginning of the pandemic. Another potential explanation may be that delaying medical care may have been a risk mitigation strategy, given that people with worse health are more likely to die from COVID-19 [13, 14]. Additionally, pandemic-related disruptions in public and personal transportation may have also impacted older adults’ ability to seek out care, especially among those with disabilities. Our study could not distinguish between these potential explanations. Nevertheless, the potential for chronic conditions to worsen without proper medical supervision makes the delays in medical care among older adults with worse health concerning and may lead to greater health differences by health status. Future research should evaluate whether the health of older adults with worse health was impacted by delays in care, and the potential explanations to provide better access and maintenance during large-scale healthcare disruptions.

Additionally, we observed notable differences across types of medical care that were delayed. Overall, 30% of older adults reported delaying any type of medical care. However, delayed dental and doctor appointments were the most frequently delayed, while surgery and filling a prescription were much less likely to be delayed. These differences have implications for understanding future health. Continued medication usage (as indicated by low prevalence of delay in filling a prescription) may indicate that people who had a prior diagnosis were able to maintain their medication regimes during this period. However, the greater delays among dental and doctor appointments may lead to worse health for conditions that would have been tested and diagnosed during a medical appointment. While this study cannot directly evaluate the impact of delayed care during COVID on older adults’ health, recent reports have shown fewer ER visits and fewer medical procedures to treat cardiovascular disease compared to 2019 [15–17]. While previous studies have evaluated the national population as whole, less is known about older adults, who may be more severely impacted overall because the increased likelihood of having health complications with age, especially from chronic conditions. Future research should evaluate whether and what kind of negative consequences older adults may have experienced during the COVID-19 pandemic, and how that may have impacted their overall health.

The study has some limitations. First, the data were collected from June to September, at a time when individuals and health care offices were adjusting to the new health guidelines resulting in temporary closures, a second peak occured in July, and the timeline of the pandemic was largely unknown; each of these conditions may have impacted decisions to delay care. Second, while we believe that risk assessment and limited healthcare appointments impacted delayed care patterns, we could not directly test these explanations. Consideration should also be given modes of access. In the HRS, respondents who replaced their physical appointments with telemedicine were not considered to have delayed care. Evaluating the use of telemedicine during this period will provide further insight into how the expansion of healthcare through technology may improve healthcare access, especially during periods of significant social disruptions. And whether telemedicine curtailed negative impacts of more limited in-person appointments. Third, beyond the broad categories of care, we could not evaluate differences by specific needs. For example, for doctor’s appointments, we could not assess whether delayed care was related to routine check-ups or more pressing medical needs. Relatedly, we could not evaluate how COVID-19-related care may have influenced these patterns; delaying medical appointments may have been even greater if COVID-19 related appointments are excluded.

## Conclusions

Our study has described the types of delayed care and the sociodemographic and health status differences in delaying care during the COVID-19 pandemic. The proportion of older adults delaying care should be a key concern for health researchers and providers. The COVID-19 pandemic not only has led to hundreds of thousands of deaths, which were concentrated among older adults, but also may have undermined health through changes in healthcare access. As healthcare usage returns to pre-pandemic levels, medical practitioners may face significant challenges in addressing the healthcare needs of the population due to increased demand from regular usage along with the catch-up procedures from delayed care. Taken together, this creates a burden on the healthcare system that may extend well beyond the COVID-19 pandemic, and it is less clear how long it will take for the healthcare system to meet these demands. Consideration should also be given to how the pandemic may have impacted health usage levels overall, such as declines in overuse. This study provides some of the first estimates of how widespread delaying medical care was among older adults, which provides insight into the potential burden on the healthcare system. Additionally, the sociodemographic and health status differences in delaying care point to the importance of future work to evaluate how the COVID-19 pandemic may have created worse health inequities among older adults beyond mortality. These differences may exacerbate already existing health inequalities. This additional consequence of the pandemic should be at the forefront of our understanding of older adult health in the United States and its role in deteriorating overall wellbeing, especially as health researchers and policy makers come to understand the full consequences of the COVID-19 pandemic.

## Supplementary Information

**Additional file 1: Supplemental Table 1.** Odds Ratios from Logistic Regression Models Predicting Delayed Care Among Older Adults 54+ During the COVID-19 Pandemic (HRS COVID Subsample 2020).

## Data Availability

The datasets used in this study are publicly available and can be assessed through the Health and Retirement Study website: https://hrsdata.isr.umich.edu/data-products/public-survey-data?_ga=2.206931923.1508667504.1634583992-1398445985.1634583992

## Abbreviations

HRS: Health and Retirement Study
OR: Odds Ratio
CI: Confidence Interval
