# Supplementary material for: Sociodemographic and health status differences in delaying medical care during the COVID-19 pandemic among older adults: findings from the Health and Retirement Study
Source: BMC Public Health. 2022 Sep 10;22:1720. doi: 10.1186/s12889-022-14118-4 (PMC9463671; doi:10.1186/s12889-022-14118-4)
Supplement: Supplementary file 1 — Additional file 1: Supplemental Table 1. Odds Ratios from Logistic Regression Models Predicting Delayed Care Among Older Adults 54+ During the COVID-19 Pandemic (HRS COVID Subsample 2020). [file 12889_2022_14118_MOESM1_ESM.docx]

| **Supplemental Table 1. Odds Ratios from Logistic Regression Models Predicting Delayed Care Among Older Adults 54+ During the COVID-19 Pandemic (HRS COVID Subsample 2020)** | | | | | | |
| --- | --- | --- | --- | --- | --- | --- |
| Age | 0.97*** | (0.96,0.98) | 0.97*** | (0.96,0.98) | 0.97*** | (0.95,.98) |
| Women (Reference: Men) | 1.35** | (1.08, 1.68) | 1.33* | (1.07,1.67) | 1.35** | (1.08,1.69) |
| Race/Ethnicity: NH Whites) |  |  |  |  |  |  |
| Non-Hispanic Blacks | 0.98 | (0.72,1.32) | 1.03 | (0.77,1.37) | 0.99 | (0.74,1.33) |
| Hispanics | 0.78 | (0.55,1.12) | 0.87 | (0.61,1.25) | 0.82 | (0.58,1.16) |
| Non-Hispanic Others | 0.76 | (0.45, 1.28) | 0.77 | (0.46,1.29) | 0.78 | (0.46,1.30) |
| Education (Reference: Less than High School) |  |  |  |  |  |  |
| High School or GED | 1.11 | (0.76, 1.60) | 1.05 | (0.73,1.53) | 1.11 | (0.76,1.61) |
| Some College | 1.24 | (0.85, 1.81) | 1.15 | (0.78,1.68) | 1.24 | (0.84,1.81) |
| Bachelor's Degree and Above | 1.79** | (1.22,2.63) | 1.68** | (1.14,2.45) | 1.80** | (1.23,2.64) |
| Cardiometabolic Conditions | |  |  |  |  |  |
| Diabetes | 1.28 | (0.99,1.64) |  |  |  |  |
| Heart Disease | 1.06 | (0.81,1.39) |  |  |  |  |
| Stroke | 0.80 | (0.53,1.21) |  |  |  |  |
| High Blood Pressure | 1.11 | (0.87,1.42) |  |  |  |  |
| Number of Doctor Visits in 2018 |  |  |  |  |  |  |
| 1 to 5 |  |  | 1.67* | (1.07,2.60) |  |  |
| 5 to 10 |  |  | 1.42 | (0.87,2.33) |  |  |
| 11+ |  |  | 1.90** | (1.17,3.08) |  |  |
| Number of Cardiometabolic Conditions (0-4) |  |  |  |  | 1.10 | (0.99,1.23) |
| Exponentiated coefficients; 95% confidence intervals in second column | | | | |  |  |
| * p<.05, ** p<.01, *** p<.001 | |  |  |  |  |  |
| **Supplemental Table 1. Odds Ratios from Logistic Regression Models Predicting Delayed Care Among Older Adults 54+ During the COVID-19 Pandemic (HRS COVID Subsample 2020)** | | | | | | |
| Age | 0.97*** | (0.96,0.98) | 0.97*** | (0.96,0.98) | 0.97*** | (0.95,.98) |
| Women (Reference: Men) | 1.35** | (1.08, 1.68) | 1.33* | (1.07,1.67) | 1.35** | (1.08,1.69) |
| Race/Ethnicity: NH Whites) |  |  |  |  |  |  |
| Non-Hispanic Blacks | 0.98 | (0.72,1.32) | 1.03 | (0.77,1.37) | 0.99 | (0.74,1.33) |
| Hispanics | 0.78 | (0.55,1.12) | 0.87 | (0.61,1.25) | 0.82 | (0.58,1.16) |
| Non-Hispanic Others | 0.76 | (0.45, 1.28) | 0.77 | (0.46,1.29) | 0.78 | (0.46,1.30) |
| Education (Reference: Less than High School) |  |  |  |  |  |  |
| High School or GED | 1.11 | (0.76, 1.60) | 1.05 | (0.73,1.53) | 1.11 | (0.76,1.61) |
| Some College | 1.24 | (0.85, 1.81) | 1.15 | (0.78,1.68) | 1.24 | (0.84,1.81) |
| Bachelor's Degree and Above | 1.79** | (1.22,2.63) | 1.68** | (1.14,2.45) | 1.80** | (1.23,2.64) |
| Cardiometabolic Conditions | |  |  |  |  |  |
| Diabetes | 1.28 | (0.99,1.64) |  |  |  |  |
| Heart Disease | 1.06 | (0.81,1.39) |  |  |  |  |
| Stroke | 0.80 | (0.53,1.21) |  |  |  |  |
| High Blood Pressure | 1.11 | (0.87,1.42) |  |  |  |  |
| Number of Doctor Visits in 2018 |  |  |  |  |  |  |
| 1 to 5 |  |  | 1.67* | (1.07,2.60) |  |  |
| 5 to 10 |  |  | 1.42 | (0.87,2.33) |  |  |
| 11+ |  |  | 1.90** | (1.17,3.08) |  |  |
| Number of Cardiometabolic Conditions (0-4) |  |  |  |  | 1.10 | (0.99,1.23) |
| Exponentiated coefficients; 95% confidence intervals in second column | | | | |  |  |
| * p<.05, ** p<.01, *** p<.001 | |  |  |  |  |  |
